# Early initiation of rivaroxaban after reperfusion therapy for stroke patients with nonvalvular atrial fibrillation

**DOI:** 10.1371/journal.pone.0264760

**Published:** 2022-04-06

**Authors:** Junpei Koge, Hiroshi Yamagami, Kazunori Toyoda, Masahiro Yasaka, Teruyuki Hirano, Toshimitsu Hamasaki, Takehiko Nagao, Shinichi Yoshimura, Masahito Fujishige, Akira Tempaku, Shinichiro Uchiyama, Etsuro Mori, Masatoshi Koga, Kazuo Minematsu

**Affiliations:** 1 Department of Cerebrovascular Medicine, National Cerebral and Cardiovascular Center, Suita, Japan; 2 Department of Stroke Neurology, National Hospital Organization Osaka National Hospital, Osaka, Japan; 3 Department of Cerebrovascular Medicine and Neurology, National Hospital Organization Kyushu Medical Center, Clinical Research Institute, Fukuoka, Japan; 4 Department of Stroke and Cerebrovascular Medicine, Kyorin University, Tokyo, Japan; 5 The George Washington University Biostatistics Center, Rockville, Maryland, United States of America; 6 Department of Neurology, Nippon Medical School Tama-Nagayama Hospital, Tokyo, Japan; 7 Department of Neurosurgery, Hyogo College of Medicine, Hyogo, Japan; 8 Department of Neurosurgery, Shinsapporo Neurosurgical Hospital, Sapporo, Japan; 9 Department of Neurosurgery, Hokuto Hospital, Obihiro, Japan; 10 Clinical Research Center for Medicine, Center for Brain and Cerebral Vessels, Sanno Medical Center, International University of Health and Welfare Director, Tokyo, Japan; 11 Department of Behavioral Neurology and Neuropsychiatry, United Graduate School of Child Development, Osaka University, Suita, Japan; Hospital Dr. Rafael A. Calderón Guardia, CCSS, COSTA RICA

## Abstract

**Background:**

The optimal timing of initiating oral anticoagulants after reperfusion therapy for ischemic stroke is unknown. Factors related to early initiation of rivaroxaban and differences in clinical outcomes of stroke patients with nonvalvular atrial fibrillation (NVAF) who underwent reperfusion therapy was investigated.

**Methods:**

From data of 1,333 NVAF patients with ischemic stroke or transient ischemic attack (TIA) in a prospective multicenter study, patients who started rivaroxaban after intravenous thrombolysis and/or mechanical thrombectomy were included. The clinical outcomes included the composite of ischemic events (recurrent ischemic stroke, TIA, or systemic embolism) and major bleeding at 3 months.

**Results:**

Among the 424 patients, the median time from index stroke to starting rivaroxaban was 3.2 days. On multivariable logistic regression analysis, infarct size (odds ratio [OR], 0.99; 95%CI, 0.99–1.00) was inversely and successful reperfusion (OR, 2.13; 95%CI, 1.24–3.72) was positively associated with initiation of rivaroxaban within 72 hours. 205 patients were assigned to the early group (< 72 hours) and 219 patients (≥ 72 hours) to the late group. Multivariable Cox regression models showed comparable hazard ratios between the two groups at 3 months for ischemic events (hazard ratio [HR], 0.18; 95%CI, 0.03–1.32) and major bleeding (HR, 1.80; 95%CI, 0.24–13.54).

**Conclusions:**

Infarct size and results of reperfusion therapy were associated with the timing of starting rivaroxaban. There were no significant differences in the rates of ischemic events and major bleeding between patients after reperfusion therapy who started rivaroxaban < 72 hours and ≥ 72 hours after the index stroke.

**Clinical trial registration:**

Unique identifier: NCT02129920; URL: https://www.clinicaltrials.gov.

## Introduction

Patients with atrial fibrillation (AF)-related acute ischemic stroke (AIS) have a risk of early recurrent ischemic stroke, reportedly between 0.4% and 1.3% per day within the first 14 days [1–3]. Since recurrent ischemic events are likely to occur during the early period after the index stroke [4], secondary prevention from the early stage after AIS is important. However, hemorrhagic transformation occurs frequently in the early stage after cardioembolic stroke [5]. Early initiation of anticoagulation has concerns about the increased risk of hemorrhagic transformation. Thus, the optimal timing of initiating oral anticoagulants (OACs) after AIS is unclear.

Direct oral anticoagulants (DOACs) have a rapid anticoagulant effect and lower risks of intracranial hemorrhage compared with vitamin K antagonists (VKAs) [6]. Early initiation of DOACs may be a promising option for patients with AF-related AIS. In clinical practice, DOACs are often started earlier after AIS than recommendations in the clinical guidelines [7]. Recent observational studies have reported the safety of early DOAC initiation [8,9]. However, these studies mainly included mild AIS patients without reperfusion therapy who had lower risks of hemorrhagic transformation. Patients who have undergone reperfusion therapy are likely to have hemorrhagic transformation due to large-sized infarction and endovascular procedure [10]. Patients after reperfusion therapy may have significantly different post-treatment conditions depending on results of reperfusion therapy, which may affect the timing of initiating anticoagulation and rates of recurrent ischemic or hemorrhagic events. The difference in clinical outcomes according to the timing of initiating anticoagulation after reperfusion therapy remains unclear.

We aimed to investigate factors related to early initiation of rivaroxaban after reperfusion therapy and assess the difference in clinical outcomes depending on the timing of initiating rivaroxaban for patients after intravenous thrombolysis (IVT) and/or mechanical thrombectomy (MT), using data from the Recurrent Embolism Lessened by rivaroxaban, an Anti-Xa agent, of Early Dosing for acute ischemic stroke and transient ischemic attack with atrial fibrillation (RELAXED) study [11,12].

## Methods

### Study subjects

The RELAXED study is a prospective, multicenter, observational study to investigate the optimal timing to start rivaroxaban and the efficacy and safety of rivaroxaban for AIS patients with nonvalvular AF (NVAF). The detailed study design and baseline data were previously published [11,12]. Participants were enrolled from 157 stroke centers in Japan between February 2014 and April 2016 and followed up for 3 months. Patients with AIS or transient ischemic attack (TIA) with NVAF were included if they met the following criteria: patients who were hospitalized or those who visited hospitals as outpatients within 48 hours of onset; infarct in the territory of the middle cerebral artery (MCA) demonstrated by diffusion weighted imaging (DWI) or TIA showing symptoms corresponding to this area with negative DWI; and on treatment with rivaroxaban that started within 30 days after the index stroke. In the present substudy of the RELAXED study, patients who underwent IVT and/or MT for the index AIS were included. The study protocol and procedures were reviewed and approved by the institutional review boards of each participating study center. This substudy was approved by institutional review board of our institute (R20024-2). Written informed consent was obtained from all patients or guardians of participants when the patients could not communicate verbally. The study was registered with ClinicalTrials.gov (NCT02419794) and the Japanese University Hospital Medical Information Network (UMIN) Clinical Trials Registry (UMIN000015273).

The treatment modalities were determined by each physician in charge. IVT was performed with alteplase at 0.6 mg/kg (approved dose in Japan) [13]. The MT procedures including stent retriever thrombectomy, direct aspiration, or a combination of both techniques were mainly used at the discretion of the operator (S1 Table). The timing of initiating rivaroxaban was at the discretion of the treating physicians. Fifteen milligrams of rivaroxaban once daily was administered if creatinine clearance was ≥ 50 mL/min, and 10mg once daily were administered if < 50mL/min, according to its approved dosage/administration schedule in Japan [14]. The patients were divided into 2 groups according to the timing of initiating rivaroxaban after symptom onset using the median days of the initiating rivaroxaban as a cutoff (early [< 72 hours initiation of rivaroxaban] and late groups [≥ 72 hours initiation of rivaroxaban]).

### Clinical data collection

The baseline clinical data for the following 36 variables were collected; age, sex, premorbid modified Rankin Scale (mRS) score, weight, blood pressure at admission, baseline NIHSS score, hypertension, diabetes mellitus, dyslipidemia, ischemic heart disease, congestive heart failure, previous stroke/TIA, prior antiplatelet therapy, prior anticoagulation therapy, baseline systolic blood pressure, laboratory data (hemoglobin, platelet count), creatinine clearance, infarct size, presence of occluded vessels, IVT, MT, successful recanalization in patients who had occluded vessels, any hemorrhagic transformation, bridging heparin, dose of rivaroxaban, the timing of initiating rivaroxaban, status at discharge and length of in hospital stay. The infarct size was measured using diffusion weighted imaging (DWI) by magnetic resonance imaging (MRI) performed within 48 hours after the index stroke. Infarct size was defined as small (< 4.0 cm^3^), medium (≥ 4.0 and < 22.5 cm^3^), and large (≥ 22.5 cm^3^). In the cohort of patients treated with IVT, recanalization status was evaluated by computed tomography angiography or magnetic resonance angiography within 24 hours from the start of IVT. Successful recanalization was defined as a primary arterial occlusive lesion recanalization score of 3 [15]. In patients treated with MT, recanalization status was assessed by the final angiography. Successful recanalization was defined as a modified Thrombolysis in Cerebral Infarction score of 2b or 3 [16]. Intracranial hemorrhage (ICH) before rivaroxaban administration was assessed according to the European Collaborative Acute Stroke Study classification [17]. An independent MRI imaging Evaluation Committee evaluated the images. Creatinine clearance was calculated using the Cockcroft-Gault Equation.

### Clinical outcomes

The primary efficacy outcome was the incidence of the composite of ischemic events (recurrent ischemic stroke, TIA or systemic embolism) within 90 days after onset of the index stroke. The primary safety outcome was the incidence of composite events including major bleeding including symptomatic ICH, parenchymal hematoma (PH) 2 with exacerbation of the NIHSS score (≥ 4), and another major bleeding event according to the criteria defined by the International Society on Thrombosis and Hemostasis, at 90 days after index stroke. The secondary outcomes were recurrent ischemic stroke, ICH, and death at 90 days. Any hemorrhagic and ischemic events or other events related to reperfusion therapy were judged by an independent event adjudication committee. These events were not included as endpoints in order to evaluate the risk of recurrent ischemic stroke and major bleeding without the effect of reperfusion therapy.

### Statistical analysis

Descriptive statistics were calculated for baseline demographic characteristics, imaging data, and clinical outcomes, comparing patients in the early and late groups. Continuous variables were presented as medians (interquartile range [IQR]). Categorical variables were presented as frequencies and percentages. Group comparisons were performed using the Wilcoxon rank-sum test or the Fisher’s exact test. Multiple logistic regression models were constructed to investigate the factors associated with early initiation of rivaroxaban. For model 1, the following variables that were previously reported to be associated with delayed anticoagulation and other potential confounders were included: age, sex, NIHSS score, infarct size, no previous history of AF, prior anticoagulation, creatinine clearance, presence of occluded vessels, IVT, MT, successful recanalization, and any hemorrhagic transformation. For model 2, variables with *P* < 0.10 on univariable logistic regression analysis were included in the model. We calculated cumulative incidences for the primary and secondary outcomes. The survival curves of the primary and secondary outcomes for patients in the early and late groups were estimated by Kaplan-Meier survival curves and compared by the log-rank test. We performed univariable and multivariable Cox proportional hazards regression analyses for each outcome. The following prespecified covariates were selected for the multivariable model: sex, age, body weight, baseline NIHSS score, CHA_2_DS_2_-VAS_c_ score after the index stroke, HAS-BLED score after the index stroke, infarct size, successful recanalization, IVT, MT, tube administration of rivaroxaban, and creatinine clearance. When fitting Cox regression to the data, Firth’s penalized likelihood approach was used. Hazard ratios (HRs) with 95% confidence intervals (CIs) were calculated. The Hosmer-Lemeshow χ^2^ statistics was calculated to evaluate the goodness-of-fit indices for each multivariable logistic model. The discrimination of the model was assessed using the c-statistics. Missing values were handled using a pairwise deletion method. All reported *P*-values are for a two-sided test and *P*-value < 0.05 was considered significant. Statistical analyses were performed using R version 3.6.1 (R Foundation for Statistical Computing, Vienna, Austria), with the packages lmtest, survey, Matching, and ggplot2.

## Results

### Patients’ characteristics

Of a total of 1333 patients registered in the RELAXED study, 424 patients who underwent IVT or MT (184 females, median age 77 [IQR, 71–84]) were included in the final analysis. The median baseline NIHSS score was 15 (IQR, 9–20), and 159 patients (40%) had large-sized infarcts. Overall, median days of rivaroxaban initiation after index stroke were 3.2 days (IQR, 1.8–6.7 days). 205 patients who started rivaroxaban earlier than 72 hours after index stroke were assigned in the early group and the other 219 patients starting rivaroxaban at 72 hours or later after onset were assigned to the late group. The study flow chart is shown in S1 Fig.

Clinical characteristics of the early and late groups are shown in Table 1. The early group had smaller infarcts, and a higher proportion of sustained AF and successful recanalization. Prior anticoagulation therapy and any hemorrhagic transformation were less common in the early group. The duration of hospital stay was shorter in the early group. The proportions of patients discharged home was higher in the early group. In the subgroup of patients who underwent MT, the proportion of successful recanalization was higher in the early group, though there were no significant differences in the MT procedures between the two groups (S1 Table).

**Table 1:** Clinical characteristics of the early and late groups.

	Early group (n = 205)	Late group = 219)	*P*-value
Age, y; median (IQR)	77 (70–84)	78 (71–84)	0.34
Female, n (%)	93 (45)	91 (42)	0.43
Premorbid mRS score, median (IQR)	0 (0–0)	0 (0–0)	0.82
Body weight, kg; median (IQR)	57 (49–66)	57 (49–65)	0.98
Baseline NIHSS score, median (IQR)	14 (8–19)	16 (9–16)	0.08
Systolic BP, mmHg; median (IQR)	134 (123–149)	130 (118–140)	0.005
Diastolic BP, mmHg; median (IQR)	77 (64–88)	73 (64–83)	0.21
Infarct size, cm^3^; median (IQR)	7.6 (2.2–21)	16.3 (5.2–52.5)	<0.001
CHA_2_DS_2_-VASc score after onset, median (IQR)	5 (4–6)	5 (4–6)	0.84
HASBLED score after onset, median (IQR)	3 (2–3)	3 (2–3)	0.50
Sustained AF, n (%)	106 (56)	94 (46)	0.03
AF diagnosis before index event, n (%)	91 (44)	95 (43)	0.85
Hypertension, n (%)	145 (72)	141 (65)	0.14
Diabetes mellitus, n (%)	32 (16)	34 (16)	>0.99
Dyslipidemia, n (%)	61 (31)	60 (28)	0.52
Ischemic heart disease, n (%)	14 (7)	12 (6)	0.69
Congestive heart failure, n (%)	20 (11)	22 (11)	>0.99
Previous stroke/TIA, n (%)	31 (15)	37 (17)	0.69
Prior anticoagulation therapy, n (%)	28 (14)	47 (21)	0.04
Prior antiplatelet therapy, n (%)	43 (21)	41 (19)	0.63
Hemoglogbin, g/dl; median (IQR)	13.3 (11.8–14.4)	12.8 (11.6–14.2)	0.05
Platelet count, ×10^3^/μL; median (IQR)	18.2 (15.3–21.5)	20 (15.8–26.3)	0.004
CrCl mL/min; median (IQR)	59.4 (47.9–78.9)	63.9 (49.6–79.1)	0.41
CrCl < 50 mL, min; n (%)	57 (28)	55 (25)	0.58
Presence of occluded vessels, n (%)	183 (89)	193 (90)	0.87
Intravenous thrombolysis, n (%)	171 (83)	168 (77)	0.09
Mechanical thrombectomy, n (%)	101 (49)	101 (46)	0.56
Successful recanalization, n (%)	133 (76)	111 (60)	<0.001
Any hemorrhagic transformation, n (%)	8 (4)	28 (13)	0.001
HI1	5 (2)	3 (1)	0.03
HI2	2 (1)	17 (8)	
PH1	1 (0)	6 (3)	
PH2	0 (0)	2 (1)	
Days from onset to rivaroxaban start, d; median (IQR)	1.7 (1.2–2.2)	6.6 (4.1–10.4)	<0.001
Rivaroxaban dose 10mg, n (%)	62 (30)	64 (29)	0.83
Tube administration, n (%)	28 (14)	47 (21)	0.04
Oral antiplatelet agents at hospital discharge, n (%)	15 (7)	25 (11)	0.18
Home discharge, n (%)	97 (48)	67 (31)	0.001
Rehabilitation, n (%)	99 (48)	131 (61)	
Other, n (%)	8 (4)	16 (7)	
Hospital stay, day; median (IQR)	19 (13–29)	27 (18–41)	<0.001

Abbreviations: IQR, interquartile range; mRS, modified Rankin Scale; NIHSS, National Institute of Health Stroke Scale; BP, blood pressure; AF, atrial fibrillation; TIA, transient ischemic attack; CrCl, creatinine clearance; HI, hemorrhagic infarction; PH, parenchymal hematoma.

### Factors associated with early initiation of rivaroxaban

**Fig 1:**
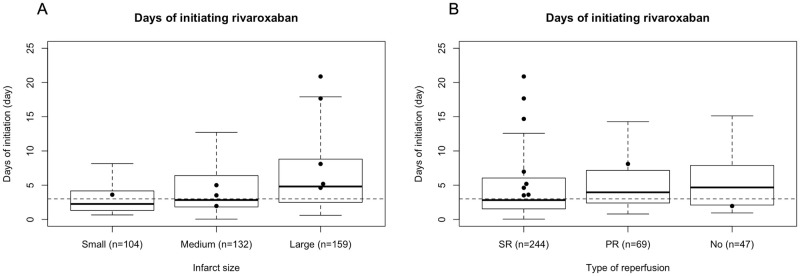
Timing of initiating rivaroxaban according to the infarct size and recanalization status. (A) Days of initiating rivaroxaban after the index stroke. The median days of starting rivaroxaban were 2.3 days (IQR, 1.3–4.2 days) in patients with small-sized infarcts, 2.8 days (IQR, 1.8–6.4 days) in patients with medium-sized infarcts, and 4.8 days in patients with large-sized infarcts (IQR, 2.5–8.9 days). (B) The median days of starting rivaroxaban were 2.8 days (IQR, 1.5–6.0 days) in patients with successful recanalization, 4.0 days (IQR, 2.4–7.2 days) in patients with partial recanalization, and 4.7 days (IQR, 2.1–7.9 days) in patients with no recanalization. Boxes represent the interquartile range. Horizontal lines across the box indicate median values, and the top and bottom edges of each box indicate the interquartile range. The whiskers represent 1.5 times the interquartile range. The dots represent the days of starting rivaroxaban in patients with ischemic stroke or transient ischemic attack or systemic embolism. Abbreviations: SR, successful recanalization, PR, partial recanalization.

Fig 1 shows the days of starting rivaroxaban after the index stroke. The median days of initiating rivaroxaban in all patients were 3.2 days (IQR, 1.8–6.7 days). The median days of initiating rivaroxaban were 2.3 days (IQR, 1.3–4.2 days) in patients with small -sized infarcts, 2.8 days (IQR, 1.8–6.4 days) in patients with medium-sized infarcts, and 4.8 days (IQR, 2.5–8.9 days) in patients with large-sized infarcts. The median days of starting rivaroxaban were 2.8 days (IQR, 1.5–6.0 days) in patients with successful recanalization, 4.0 days (IQR, 2.4–7.2 days) in patients with partial recanalization, and 4.7 days (IQR, 2.1–7.9 days) in patients with no recanalization.

On multivariable logistic regression analysis, infarct size was inversely associated with the early initiation of rivaroxaban (adjusted OR, 0.99, 95%CI, 0.99 to 1.00 per 1-cm^3^, model 1 in Table 2). Systolic blood pressure (adjusted OR, 1.27, 95%CI, 1.11 to 1.46 per 10-mmHg, model 1 in Table 2) and successful recanalization (adjusted OR, 2.13, 95%CI, 1.24 to 3.72, model 1 in Table 2) were positively associated with the early initiation of rivaroxaban. The initiation of rivaroxaban tended to be delayed for patients with any hemorrhagic transformation and those with prior anticoagulation. S2 Table shows the details of prior anticoagulation. The leading anticoagulants prescribed before onset was warfarin (71%). The median PT-INR on patients with prior warfarin was 1.2 (IQR, 1.1–1.3).

**Table 2:** Predictors of early initiation of rivaroxaban.

	Univariable		Multivariable (Model 1)^†^		Multivariable (Model 2)‡	
	OR (95% CI)	*P* value	OR (95% CI)	*P* value	OR (95% CI)	*P* value
Age (per 5 y increase)	0.96 (0.86 to 1.06)	0.40	0.83 (0.68 to 1.01)	0.06		
Male	0.86 (0.58 to 1.26)	0.43	0.71 (0.43 to 1.17)	0.18		
Systolic BP (per 10 mmHg increase)	1.18 (1.06 to 1.31)	0.003	1.27 (1.11 to 1.46)	<0.001	1.17 (1.02 to 1.34)	0.03
Baseline NIHSS score	0.98 (0.95 to 1.00)	0.07	0.99 (0.95 to 1.03)	0.49	1.00 (0.96 to 1.03)	0.90
Infarct size	0.99 (0.99 to 1.00)	<0.001	0.99 (0.99 to 1.00)	0.01	0.99 (0.99 to 1.00)	0.03
Hemoglobin	1.11 (1.01 to 1.23)	0.03			1.12 (0.99 to 1.28)	0.07
Platelet	1.00 (1.00 to 1.00)	0.40				
Sustained AF	1.54 (1.04 to 2.30)	0.03			1.58 (0.95 to 2.65)	0.08
AF diagnosed before index event	0.96 (0.65 to 1.41)	0.83	0.54 (0.31 to 0.94)	0.03		
Prior anticoagulation	0.58 (0.34 to 0.96)	0.04	0.36 (1.37 to 5.73)	0.005	0.52 (0.26 to 1.01)	0.06
Creatinine clearance	1.00 (0.99 to 1.01)	0.69	0.99 (0.98 to 1.00)	0.14		
Presence of occluded vessels	0.95 (0.51 to 1.78)	0.87	1.82 (0.66 to 5.19)	0.25		
Intravenous thrombolysis	1.53 (0.95 to 2.49)	0.09	1.90 (1.00 to 3.64)	0.05	1.29 (0.70 to 2.36)	0.41
Mechanical thrombectomy	1.13 (0.77 to 1.66)	0.52	1.90 (1.01 to 3.60)	0.05		
Successful recanalization	2.19 (1.39 to 3.48)	<0.001	2.13 (1.24 to 3.72)	0.007	2.44 (1.42 to 4.28)	0.001
Any hemorrhagic transformation	0.28 (0.85 to 1.26)	0.002	0.37 (0.12 to 1.02)	0.07	0.39 (0.14 to 0.98)	0.05

^**†**^Model 1: Adjusted for prespecified variables: age, sex, baseline NIHSS score, infarct size, previous history of AF, prior anticoagulation, creatinine clearance, presence of occluded vessels, intravenous thrombolysis, endovascular therapy, successful recanalization, hemorrhagic infarction. The model showed a c-statistic of 0.72 and a Hosmer-Lemeshow chi-squared statistic of 12.4 (*P* = 0.13).

^**‡**^Model 2: Adjusted for variables with *P*<0.1 on univariable models. The model showed a c-statistic of 0.73 and a Hosmer-Lemeshow chi-squared statistic of 5.00 (*P* = 0.76).

Abbreviations: OR, odds ratio; CI, confidence interval; BP, blood pressure; NIHSS, National Institute of Health Stroke Scale; AF, atrial fibrillation.

### Clinical outcomes

**Fig 2:**
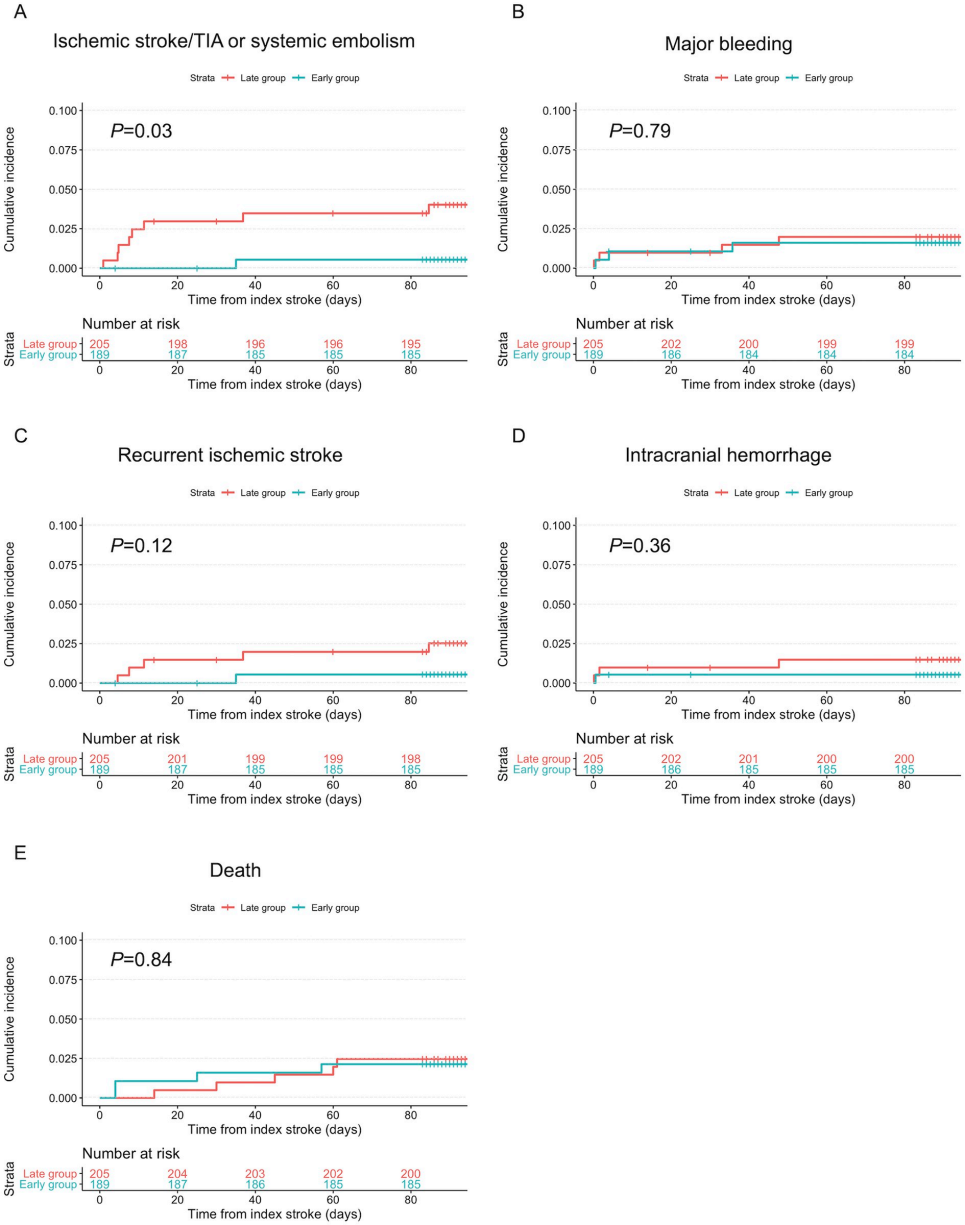
Kaplan-Meier curves for ischemic stroke or TIA or systemic embolism (A), major bleeding (B), recurrent ischemic stroke (C), intracranial hemorrhage (D), and death (E) according to the timing of starting rivaroxaban. Abbreviations: TIA, transient ischemic attack.

In total, 394 patients had available information at 3-month follow-up regarding clinical events since discharge. The total follow-up time was 124 patient-years (median follow-up, 103 days; IQR, 93–118 days). The follow-up time was 58 patient-years in the early group, and 66 patient-years in the late group. Table 3 shows the cumulative incidence of primary and secondary endpoints. In the present cohort, 6 ischemic strokes, 1 TIAs, 1 systemic embolism, 1 PH2, 2 extracranial major bleeding events, 4 intracranial hemorrhages, and 9 deaths occurred. The causes of death in patients in the early group were recurrent ischemic stroke in 1 patient, hemorrhagic infarction in 1 patient, infection in 1 patient, and unknown in 1 patient. The causes of death in patients in the late group were heart failure in 4 patients and unknown in 1 patient. Fig 2 shows the unadjusted Kaplan-Meier curves for the composite of ischemic events, major bleeding, recurrent ischemic stroke, intracranial hemorrhage, and death. Using the log-rank test, the frequency of the composite of ischemic events was significantly lower in the early group (0.5%) than in the late group (3.9%) (*P* = .03). Composite ischemic events occurred at a median of 8.3 days after the index stroke. The frequencies of major bleeding, recurrent ischemic stroke, intracranial hemorrhage, and death were not significantly different between the groups. The univariable and multivariable Cox models showed no significant differences between the early and late group in the risks for the composite of ischemic events, major bleeding, recurrent ischemic stroke, ICH, and death (Table 3).

**Table 3:** Efficacy and safety outcomes of the early and late groups.

	Cumulative incidence (Total number)	Annualized rate	Crude HR (95% CI)	Adjusted HR* (95% CI)
**Recurrent ischemic Stroke/TIA or systemic embolism**				
All patients (n = 394)	2.3% (9)	7.4%	–	–
Early group (n = 189)	0.5% (1)	1.7%	0.19 (0.03 to 1.18)	0.18 (0.03 to 1.32)
Late group (n = 205)	3.9% (8)	12.5%	1 (reference)	1 (reference)
**Major bleeding**				
All patients (n = 394)	1.8% (7)	5.7%	–	–
Early group (n = 189)	1.5% (3)	4.9%	0.84 (0.19 to 3.75)	1.80 (0.24 to 13.54)
Late group (n = 205)	1.8% (4)	6.2%	1 (reference)	1 (reference)
**Recurrent ischemic stroke**				
All patients (n = 394)	1.5% (6)	4.9%	–	–
Early group (n = 189)	0.5% (1)	1.7%	0.29 (0.04 to 2.07)	0.43 (0.05 to 3.87)
Late group (n = 205)	2.4% (5)	7.7%	1 (reference)	1 (reference)
**Intracranial hemorrhage**				
All patients (n = 394)	1.0% (4)	3.3%	–	–
Early group (n = 189)	0.5% (1)	1.7%	0.46 (0.05 to 3.94)	0.75 (0.04 to 13.00)
Late group (n = 205)	1.4% (3)	4.6%	1 (reference)	1 (reference)
**Death**				
All patients (n = 394)	2.3% (9)	7.3%	–	–
Early group (n = 189)	2.0% (4)	6.9%	0.89 (0.24 to 3.32)	1.09 (0.21 to 5.62)
Late group (n = 205)	2.3% (5)	7.7%	1 (reference)	1 (reference)

*Adjustment for age, sex, body weight, CHA_2_DS_2_–VASc score, HAS–BLED, NIHSS, infarct size, creatinine clearance, successful recanalization, any hemorrhagic transformation, endovascular therapy, intravenous thrombolysis, and tube administration.

Abbreviations: HR, hazard ratio; CI, confidence interval; TIA, transient ischemic attack.

## Discussion

The major findings of the present sub-study of the RELAXED study involving patients who underwent reperfusion therapy were: (1) infarct size was inversely associated, and successful recanalization was positively associated with the early initiation of rivaroxaban; (2) the incidence of the composite of ischemic stroke, TIA or systemic embolism at 90 days in patients starting rivaroxaban less than 72 hours after the index stroke (annualized rate, 1.7%) was relatively low as compared to those starting it at 72 hours or later (12.5%), though the difference was not significant on multivariable analysis; and (3) there were no significant differences in the incidences of major bleeding, ICH, and death.

In the present cohort, the baseline NIHSS scores and the proportion of patients with large-sized infarction were higher than in the respective studies [8]. The CHA_2_DS_2-_VAS_c_ and HASBLED score were comparable to previous retrospective observational studies of early initiation of DOACs [8,9], and the median days of initiating DOAC (3.2 days) were earlier than those of previous studies (4–5 days) [8,9]. The rate of recurrent AIS (4.9%/patient-year) was comparable to these studies (recurrent ischemic stroke or TIA of the SAMURAI-NVAF registry, 5.6%/patient-year; recurrent AIS in the NOACISP registry, 7.7%/patient-year) [8,9]. The cumulative incidences of ICH (3.3%/patient-year) were higher than the incidences in these studies (0.6–1.3%/patient-year). Since the risk of hemorrhagic transformation increases with the size of the infarction [18], the present cohort might have a higher risk of ICH. However, recurrent ischemic events were 2 times more frequent than ICH.

Recanalization status was associated with the timing of initiating DOACs in addition to factors that support delayed initiation of OAC, including size of infarction, blood pressure, and hemorrhagic transformation [19]. The timing of initiating DOACs for patients without successful recanalization may be delayed because of severe neurological conditions, dysphagia, and concern about hemorrhagic transformation due to a large-sized infarction. It is conceivable that the early group included more patients with successful recanalization and small-sized infarction, reflecting good post-treatment conditions of the early group. In patients who underwent reperfusion therapy, there can be a greater difference between the early and late group in conditions after treatment than that in the baseline.

In the present subjects, the difference in the incidence of recurrent ischemic events between early and late groups was larger than in the previous studies, though it was not statistically significant [8,9]. Rivaroxaban was administered later to patients with larger infarction and more severe neurological conditions, which was recognized as a risk factor of recurrent ischemic events [18]. The difference between the early and late group of the present subjects may be greater than that in previous studies, resulting in the larger difference of recurrent ischemic events between the early and late group. Unmeasured post-treatment conditions after reperfusion therapy in the late group may also contribute to the increased risk of recurrent ischemic events (e.g. worse blood pressure control and infection).

In the present study, early initiation of DOAC was not associated with an increased risk of major bleeding and ICH, consistent with previous studies [8,9]. The present cohort had a lower frequency of post-MT hemorrhagic transformation compared to the previous observational study of MT [10]. Moreover, the prevalence of hemorrhagic transformation before initiation of rivaroxaban was especially low in the early group. Thus, the incidence of hemorrhagic complications in the present cohort should be carefully interpreted. The present result indicates that early initiation of rivaroxaban may not be associated with an increased risk of bleeding complications even in patients after reperfusion therapy if they achieved successful reperfusion without hemorrhagic transformation. Several ongoing randomized trials to determine the appropriate timing of DOAC initiation after stroke, such as ELAN (Early Versus Late Initiation of Direct Oral Anticoagulants in Post-Ischaemic Stroke Patients With Atrial Fibrillation, NCT03148457), OPTIMAS (Optimal Timing of Anticoagulation After Acute Ischaemic Stroke, NCT03759938), TIMING (Timing of Oral Anticoagulant Therapy in Acute Ischemic Stroke With Atrial Fibrillation, NCT02961348), and START (Optimal Delay Time to Initiate Anticoagulation After Ischemic Stroke in Atrial Fibrillation, NCT03021928) will provide additional insight on the timing of DOAC initiation for stroke patients after reperfusion therapies.

The present study has several limitations other than the inherent bias due to the observational study with small sample size. First, the timing of initiating rivaroxaban was determined by each physician in charge. Thus, there might be unmeasured factors that affected the physician’s determination of the timing of initiating rivaroxaban treating physicians. These unmeasured reasons for determination might represent residual confounding factors that might have affected the results. Second, the proportion of hemorrhagic transformation was relatively low, though only patients who underwent reperfusion therapy were included. Thus, the present results cannot be generalized to AIS patients after reperfusion therapy at any level of severity. Finally, the approved dose of rivaroxaban in Japan is different from that in other countries (the standard dose of rivaroxaban in Japan, 15 md/day; reduced dose, 10 mg/day), though the pharmacokinetic and pharmacodynamic profiles of rivaroxaban 15/10mg in Japanese patients were comparable to those of 20/15mg in Caucasian populations [20].

## Conclusions

In patients after reperfusion therapy, infarct size and recanalization status after treatment was associated with the timing of starting oral anticoagulants. Consequently, there were no significant differences in the rates of ischemic events and major bleeding between patients after reperfusion therapy who started rivaroxaban earlier than 72 hours and those from 72 hours or later after the index stroke. In patients after reperfusion therapy who achieved successful recanalization without a large-sized infarction, early rivaroxaban initiation after the index stroke may be safe.

## Supporting information

S1 Checklist TREND statement checklist. (PDF)

S1 Fig Study flow chart. (DOCX)

S1 Table Details of endovascular procedures. (DOCX)

S2 Table Details of prior anticoagulation therapy. (DOCX)

S1 Dataset (XLSX)
