# Supplementary material for: Early initiation of rivaroxaban after reperfusion therapy for stroke patients with nonvalvular atrial fibrillation
Source: PLoS One. 2022 Apr 6;17(4):e0264760. doi: 10.1371/journal.pone.0264760 (PMC8985957; doi:10.1371/journal.pone.0264760)
Supplement: S1 Fig — (DOCX) [file pone.0264760.s002.docx]

**S1 Figure. Study flow chart**

Abbreviations: RELAXED, Recurrent Embolism Lessened by rivaroxaban, an Anti-Xa agent, of Early Dosing for acute ischemic stroke and transient ischemic attack with atrial fibrillation; TIA, transient ischemic attack
