# Supplementary material for: Early initiation of rivaroxaban after reperfusion therapy for stroke patients with nonvalvular atrial fibrillation
Source: PLoS One. 2022 Apr 6;17(4):e0264760. doi: 10.1371/journal.pone.0264760 (PMC8985957; doi:10.1371/journal.pone.0264760)
Supplement: S1 Table — (DOCX) [file pone.0264760.s003.docx]

**S1 Table. Details of endovascular procedures**

|  | All patients  (n = 202) | Early group  (n = 101) | Late group  (n = 101) | *P*-value |
| --- | --- | --- | --- | --- |
| Stent retriever alone, n (%) | 81 (40) | 47 (47) | 34 (34) | 0.08 |
| Contact aspiration alone, n (%) | 68 (34) | 34 (34) | 34 (34) | >0.99 |
| Both stent retriever and aspiration, n (%) | 37 (18) | 14 (14) | 23 (23) | 0.14 |
| Balloon angioplasty, n (%)* | 9 (4) | 4 (4) | 5 (5) | >0.99 |
| Intra-arterial thrombolysis, n (%)* | 9 (4) | 3 (3) | 6 (6) | 0.50 |
| Other procedures, n (%)* | 9 (4) | 5 (5) | 4 (4) | >0.99 |
| Complete recanalization, n (%) | 151 (75) | 85 (84) | 66 (65) | 0.003 |
| Puncture to recanalization time, median (IQR), min | 60 (40–91) | 55 (41–86) | 60 (40–96) | 0.49 |

*Some of these procedures were performed in combination with other mechanical thrombectomy procedures.

Abbreviations: IQR, interquartile range
