# Supplementary material for: Early initiation of rivaroxaban after reperfusion therapy for stroke patients with nonvalvular atrial fibrillation
Source: PLoS One. 2022 Apr 6;17(4):e0264760. doi: 10.1371/journal.pone.0264760 (PMC8985957; doi:10.1371/journal.pone.0264760)
Supplement: S2 Table — (DOCX) [file pone.0264760.s004.docx]

**S2 Table I. Details of prior anticoagulation**

|  | All patients  (n = 424) | Early group  (n = 205) | Late group  (n = 219) | *P*-value |
| --- | --- | --- | --- | --- |
| Warfarin, n (%) | 53 (13) | 20 (10) | 33 (15) | 0.11 |
| Dabigatran, n (%) | 9 (2) | 3 (1) | 6 (3) | 0.51 |
| Rivaroxaban, n (%) | 9 (2) | 3 (1) | 6 (3) | 0.51 |
| Apixaban, n (%) | 4 (1) | 2 (1) | 2 (1) | >0.99 |
